# Anti-Tn Monoclonal Antibody Attenuates Hyperoxia-Induced Lung Injury by Inhibiting Oxidative Stress and Inflammation in Neonatal Mice

**DOI:** 10.3389/fphar.2020.568502

**Published:** 2020-09-08

**Authors:** Chung-Ming Chen, Jaulang Hwang, Hsiu-Chu Chou, Chinde Chen

**Affiliations:** ^1^ Department of Pediatrics, Taipei Medical University Hospital, Taipei, Taiwan; ^2^ Department of Pediatrics, School of Medicine, College of Medicine, Taipei Medical University, Taipei, Taiwan; ^3^ Taipei Cancer Center, Taipei Medical University, Taipei, Taiwan; ^4^ Department of Anatomy and Cell Biology, School of Medicine, College of Medicine, Taipei Medical University, Taipei, Taiwan; ^5^ Department of Research and Development, Taivital Biopharmaceutical Co. LTD, Taoyuan, Taiwan

**Keywords:** anti-Tn monoclonal antibody, cytokine, hyperoxia, mean linear intercept, nuclear factor-*κ*B

## Abstract

Maternal immunization with Tn vaccine increases serum anti-Tn antibody titers and attenuates hyperoxia-induced lung injury in neonatal rats. This study determined whether anti-Tn monoclonal antibody can protect against hyperoxia-induced lung injury in neonatal mice. Newborn BALB/c mice were exposed to room air (RA) or normobaric hyperoxia (85% O_2_) for 1 week, creating four study groups as follows: RA + phosphate-buffered saline (PBS), RA + anti-Tn monoclonal antibody, O_2_ + PBS, and O_2_ + anti-Tn monoclonal antibody. The anti-Tn monoclonal antibody at 25 μg/g body weight in 50 μl PBS was intraperitoneally injected on postnatal days 2, 4, and 6. Hyperoxia reduced body weight and survival rate, increased mean linear intercept (MLI) and lung tumor necrosis factor-α, and decreased vascular endothelial growth factor (VEGF) expression and vascular density on postnatal day 7. Anti-Tn monoclonal antibody increased neonatal serum anti-Tn antibody titers, reduced MLI and cytokine, and increased VEGF expression and vascular density to normoxic levels. The attenuation of lung injury was accompanied by a reduction in lung oxidative stress and nuclear factor-*κ*B activity. Anti-Tn monoclonal antibody improves alveolarization and angiogenesis in hyperoxia-injured newborn mice lungs through the suppression of oxidative stress and inflammation.

## Introduction

Oxygen treatment is commonly mandatory to treat newborn infants with respiratory distress. Nevertheless, newborn infants with respiratory failure treated with supplemental oxygen displayed increased oxidant stress and resulted in lung injury. Neonatal rodent exposed to prolonged hyperoxia exhibited impaired alveolarization and angiogenesis and increased inflammation that is comparable to human bronchopulmonary dysplasia (BPD) (Berger and Bhandari, 2014; Nardiello et al., 2017). The pathogenesis of BPD is multifactorial and characterized by the arrest of alveolar and vascular growth associated with inflammation (Baraldi and Filippone, 2007; Thébaud and Kourembanas, 2017; Bonadies et al., 2020). BPD is still an important reason of morbidity and mortality in preterm infants even with ideal ventilation policies, increased use of noninvasive ventilation, and early administration of surfactant (Principi et al., 2018). Many infants with BPD left with significant respiratory morbidity, including reactive airways dysfunction and the development of obstructive lung disease during childhood (Northway et al., 1990; Jacob et al., 1998; Gien and Kinsella, 2011).

Tn antigen is expressed in bladder, colon, breast, lung, and pancreas carcinomas and uncommonly in blood cancers (Springer, 1997; Desai, 2000). Tn antigen is one of the outstanding carbohydrate antigens associated with tumor and is *α*-linked to a serine or threonine residue (Zhang et al., 2005). Tn antigen is N-acetylgalactosamine (GalNAc) *α*-linked to a serine or threonine residue, which is one of the most remarkable tumor-associated carbohydrate antigens (Zhang et al., 2005). Tn has been related to immune diseases besides cancers. Chronic inflammatory tissue in rheumatoid arthritis or osteoarthritis patients exhibits Tn antigen (Ishino et al., 2010). Elevated Tn antigen expression is observed in inflammation-inflicted tissue damage (Fu et al., 2011; Mazurov et al., 2012; Lin et al., 2014) and in hyperoxia-exposed adult murine lung, and Tn immunization protects against hyperoxia-induced lung injury through inhibition of the nuclear factor-*κ*B (NF-*κ*B) (Chen et al., 2018).

We revealed that maternal immunization with Tn vaccine increased neonatal rat serum levels of anti-Tn antibody; reduced mean linear intercept (MLI), lung cytokine stress, and oxidative stress; and increased vascular density and growth factor expressions to normoxic levels in hyperoxia-injured neonatal rats (Chen et al., 2019). These findings suggest that anti-Tn antibody has therapeutic effects on rats with hyperoxia-induced lung injury. At present, there is no available clinical treatment to prevent the development of BPD in preterm infants notwithstanding the efforts to decrease BPD (Saugstad et al., 2019). Therefore, we hypothesize that anti-Tn monoclonal antibody treatment could attenuate hyperoxia-induced lung injury in neonatal mice. The present study represents an effort to evaluate whether anti-Tn monoclonal antibody can protect against hyperoxia-induced lung injury. In addition, we further clarified the mechanisms underlying the protective effects.

## Materials and Methods

### Animal Model

All experimental procedures were approved by the Animal Care and Use Committee at Taipei Medical University (LAC-2019-0149) and performed in accordance with institutional guidelines. Timed-pregnant BALB/c mice were allowed to deliver pups vaginally at term. Within 12 h of birth, litters were pooled and randomly redistributed to the newly delivered mothers and then exposed to either hyperoxia (O_2_) or RA, creating four study groups as follows: RA + PBS, O_2_ + PBS, RA + anti-Tn monoclonal antibody, and O_2_ + anti-Tn monoclonal antibody. The nursing mothers were rotated between O_2_ treatment and RA control litters every 24 h to avoid oxygen toxicity in the mothers and eliminate maternal effects between the treatment subgroups. The oxygen rich atmosphere was maintained in a transparent 40 × 50 × 60 cm^3^ plexiglass chamber that received O_2_ continuously at 4 L/min. The oxygen levels were monitored using a ProOx Model 110 monitor (NexBiOxy, Hsinchu, Taiwan). The hyperoxia groups were placed in an environment with 85% O_2_ for 1 week. The RA groups were kept in a normoxic environment for 1 week. Survival rates were recorded daily. Body weights were recorded at the time of death. On postnatal day 7, mice pups were anesthetized with 1% isoflurane (Halocarbon Laboratories, River Edge, NJ, USA) and weighed. Blood was withdrawn from the heart to determine anti-Tn antibody levels. Right middle lobe of the right lung was obtained for histological analysis (morphometry and vascular density), and the rest lung tissues were used for biochemical analysis (vascular endothelial growth factor (VEGF) and cytokine).

### Anti-Tn Monoclonal Antibody Preparation

Anti-Tn monoclonal antibody was generated as described from another group (Chiang et al., 2012). Briefly, we conjugated Tn to a bipartite antigen carrier protein and then used the protein conjugate to immunize BALB/c mice. Isolated spleen cells from the immunized mice were fused with myeloma cells (Sp2/0-Ag14, purchased from BCRC) using a standard hybridoma technique. The generated hybridoma cells were monitored using an inverted light microscope, and the anti-Tn monoclonal antibody-positive clones were screened using an enzyme-linked immunosorbent assay (ELISA). For ascites preparation, the female BALB/c mice were preinjected intraperitoneally with 0.5 ml of pristane (Sigma-Aldrich, St. Louis, MO, USA) 1 and 3 days prior to the inoculation of 5 × 10^5^ hybridoma cells in 0.1 ml 0.9% saline solution. The ascites fluid was clarified by centrifuging at 13,200 rpm for 5 min at room temperature, and the supernatant was collected through a 0.22-µm sterile filter. The ascites and protein G resin (GenScript, Piscataway, NJ, USA) were incubated with PBS at 4°C overnight. We resuspended the resin and ascites into a column. The column was washed with PBS and further washed with PBS containing 500 mM NaCl. Subsequently, the antibody was eluted with elution buffer (200 mM Glycine, pH 2.5). The elution was collected and immediately neutralized to pH 7.4 using neutralization buffer (1 M Tris-HCl, pH 9.0).

### Administration of Anti-Tn Monoclonal Antibody

The anti-Tn monoclonal antibody-treated groups were intraperitoneally injected with anti-Tn monoclonal antibody at 25 μg/g of body weight in 50 μl PBS on postnatal days 2, 4, and 6. The PBS-treated groups were treated with the same amount of PBS at the same times. The dosages were not defined in the murine pups exposed to postnatal hyperoxia. Our goal was to administer anti-Tn monoclonal antibody to determine its beneficial effects on lung inflammation and development. Based on this concern, an appropriately high dose was selected in this study.

### Measurement of the Remaining Injected Anti-Tn Monoclonal Antibody in Serum

The levels of the injected anti-Tn monoclonal antibody remaining in serum were measured by an ELISA (SpectraPlate-96 HB, PerkinElmer PerkinElmer, Alameda, CA, USA). We dissolved 2 µg Tn antigen in 1 ml of coating buffer (14 mM sodium carbonate, 35 mM sodium bicarbonate, pH 9.6). An aliquot of 100 µl was added to each well, and the plates were incubated at 4°C overnight. After being washed three times with PBS, the plates were blocked with 200 µl of blocking buffer per well for 2 h at 37°C. After three washings with PBS, 100 µl of diluted serum (the dilutions used were 1:2,000 and 1:4,000) was added to each well and incubated at 4°C overnight. After the plates were washed three more times with PBS, 100 µl of HRP-conjugated goat anti-mouse IgG (1:5,000 dilution; Southern Biotech, Birmingham, AL, USA) was added to each well. The plates were then further incubated at 37°C for 1 h. Finally, after another three washes with PBS, 100 µl of tetramethylbenzidine substrate buffer (BD OptEIA™, San Jose, CA, USA) was added to each well followed by incubation in the dark for 5 min or longer. The color reaction was stopped by adding 50 µl of 1N HCl. The absorbance was read at a wavelength of 450 nm using a microplate spectrophotometer (Epoch, BioTek, VT, USA).

### Western Blot Analysis of VEGF

Lung tissues were homogenized in ice-cold buffer, sonicated, and centrifuged to remove cellular debris. 30 μg proteins were resolved and electroblotted to a polyvinylidene difluoride membrane (ImmobilonP, Millipore, Bedford, MA, USA). The membranes were incubated with antibody against VEGF (Santa Cruz Biotechnology) and anti-*β*-actin (Sigma-Aldrich, St. Louis, MO, USA) and subsequently with horseradish peroxidase-conjugated goat antimouse IgG (Pierce Biotechnology, Rockford, IL, USA) after being blocked with 5% nonfat dry milk. We used SuperSignal Substrate from Pierce Biotechnology to detect protein bands. Densitometric analysis was performed using AIDA software to measure the intensity of VEGF and *β*-actin bands.

### Western Blot Analysis of NF-*κ*B

We extracted nuclear proteins to detect NF-*κ*B p65 (Santa Cruz Biotechnology, CA, USA). Protein concentrations were determined using a bicinchoninic acid protein assay kit. After the membranes were being blocked with 5% skim milk at room temperature for 1 h, proteins were separated on a 12% sodium dodecyl sulfate polyacrylamide gel and transferred onto polyvinylidene difluoride membranes. The membranes were incubated with HRP-conjugated secondary antibody at room temperature for 1 h. We visualized the signal using enhanced chemiluminescence reagents according to the manufacturer’s protocol. Antibodies to *β*-actin were used as internal controls for nuclear protein loading.

### Lung TNF-α

Lung tumor necrosis factor-α (TNF-α) levels were determined using the Bio-Plex multiplex assay system (Bio-Rad, Hercules, CA, USA) and Procarta immunoassay kit (Affymetrix), according to the manufacturer’s protocol.

### Lung 8-OHdG Measurement

Lung 8-hydroxy-2′-deoxyguanosine (8-OHdG) levels were measured using an ELISA kit (BioVision, Milpitas, CA, USA), according to the manufacturer’s protocol.

### Histological Evaluation

The lungs of the neonatal mice were placed in 4% paraformaldehyde overnight. The lungs were washed in PBS and serially dehydrated in increasing concentrations of ethanol before embedment in paraffin. To standardize analysis, sections were taken from the right middle lobe of the right lung. We stained 5-μm lung tissue sections with hematoxylin and eosin, examined them using light microscopy, and assessed them for lung morphometry and fibrosis. MLI, an indicator of mean alveolar diameter, was assessed in 10 nonoverlapping fields (Chou et al., 2016). Vascular density was determined in an unbiased manner in a minimum of four random lung fields stained with von Willebrand factor (vWF). Microvessel density was determined by counting the vessels with the positive vWF stained in an unbiased manner and a minimum of four random lung fields at ×400 magnifications (Irwin et al., 2007).

### Immunohistochemistry of Lung Tissue

Immunohistochemical staining was performed on 5-μm-thick paraffin sections using immunoperoxidase visualization. After routine deparaffinization, heat-induced epitope retrieval was performed by immersing the slides in 0.01 M sodium citrate buffer (pH 6.0). The sections were then incubated for 20 h at 4°C with mouse monoclonal anti-8-OHdG antibody (Abcam, Cambridge, MA, USA), anti-NF-*κ*B-p65 (Santa Cruz Biotechnology), rabbit polyclonal anti-vWF antibody (Abcam), or rabbit polyclonal anti-VEGF antibody (Santa Cruz Biotechnology) as primary antibodies. Sections were the washed in PBS for 10 min at three times and then incubated with the suitable fluorochrome-labeled secondary antibody after being probed with the primary antibody. Each selected frame was a representative image obtained from serial confocal Z-sections (where the Z-interval between two sections was 0.2 µm) within a stack of 20 sections.

### Statistical Analysis

All data were presented as mean ± SD. Comparison of the data was performed with two-way analysis of variance and Bonferroni *post-hoc* testing. The survival rate was evaluated using the Kaplan–Meier analysis. A p value < 0.05 was considered statistically significant.

## Results

Eight dams gave birth to a total of 64 pups; 31 and 33 pups were randomly distributed to RA and hyperoxia groups, respectively. Next, 19 and 12 pups and 20 and 13 pups were treated with PBS and anti-Tn monoclonal antibody in the RA and hyperoxia groups, respectively.

### Anti-Tn Monoclonal Antibody Increased the Survival Rate of Hyperoxia-Exposed Neonatal Mice

**Figure 1:**
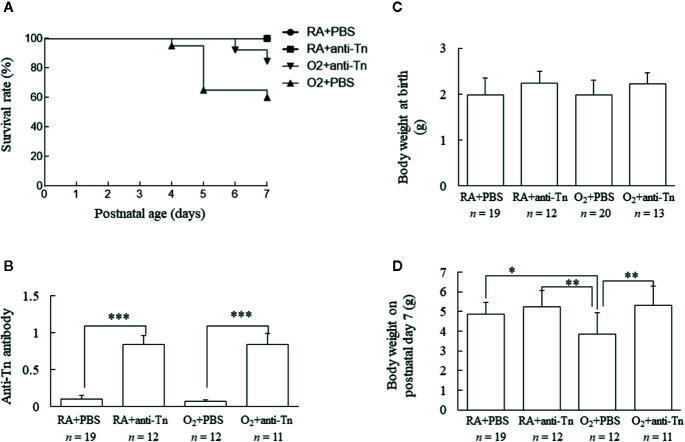
Anti-Tn monoclonal antibody increased the survival rate, serum anti-Tn antibody levels, and the body weight of hyperoxia-exposed neonatal mice on postnatal day 7. **(A)** Kaplan–Meier survival curve of mice. Anti-Tn monoclonal antibody increased the survival rate of hyperoxia-exposed neonatal mice. **(B)** Neonatal serum anti-Tn monoclonal antibody levels analyzed by ELISA. Anti-Tn monoclonal antibody treatment on postnatal days 2, 4, and 6 significantly increased serum anti-Tn monoclonal antibody levels compared with PBS treatment in RA- and O_2_-reared mice. **(C, D)** Body weights at birth and on postnatal day 7. Body weights were comparable among four study groups at birth. The mice in the hyperoxia + PBS group exhibited a significantly lower body weight on postnatal day 7 than those in the RA + PBS and RA + anti-Tn monoclonal antibody groups. Anti-Tn monoclonal antibody treatment increased the body weight on postnatal day 7 of mice reared in hyperoxia. Data presented as mean ± SD. *p < 0.05, **p < 0.01, ***p < 0.001. RA, room air; PBS, phosphate-buffered saline.

The mice reared in RA and treated with PBS or anti-Tn monoclonal antibody all survived (**Figure 1A**). The mice reared in hyperoxia and receiving PBS exhibited a lower survival rate after postnatal day 5. On postnatal day 7, the mice reared in hyperoxia and treated with anti-Tn monoclonal antibody exhibited a significantly higher survival rate compared with the mice reared in hyperoxia and treated with PBS.

### Anti-Tn Monoclonal Antibody Increased Serum Anti-Tn Antibody Levels

Serum anti-Tn antibody levels were measured on postnatal day 7 and as shown in **Figure 1B**. Serum anti-Tn antibody levels were low in mice reared in RA or a hyperoxia-inducing environment and treated with PBS (**Figure 1B**). Anti-Tn monoclonal antibody treatment on postnatal days 2, 4, and 6 substantially increased the serum levels of anti-Tn monoclonal antibody compared with PBS treatment in RA- and O_2_-reared mice on postnatal day 7.

### Anti-Tn Monoclonal Antibody Increased the Body Weight of Hyperoxia-Exposed Neonatal Mice

Body weights were comparable among the four study groups at birth (**Figure 1C**). The mice in the hyperoxia + PBS group exhibited a significantly lower body weight on postnatal day 7 than those in the RA + PBS or RA + anti-Tn monoclonal antibody group. Anti-Tn monoclonal antibody treatment increased the body weight (on postnatal day 7) of mice reared in hyperoxia (**Figure 1D**).

### Anti-Tn Monoclonal Antibody Improved Alveolarization in Hyperoxia-Injured Neonatal Mice

**Figure 2:**
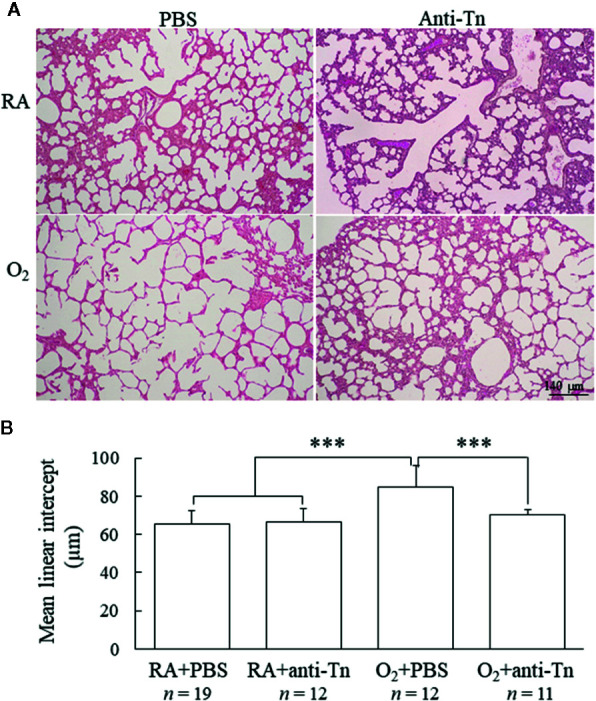
Anti-Tn monoclonal antibody improved alveolarization in hyperoxia-injured neonatal mice. **(A)** Representative hematoxylin- and eosin-stained lung sections and **(B)** MLI in the lung tissue of 7-day-old mice. The mice reared in hyperoxia and treated with PBS exhibited larger alveoli and significantly higher MLI than did those reared in RA and treated with PBS or anti-Tn monoclonal antibody. Treatment with anti-Tn monoclonal antibody significantly decreased the hyperoxia-induced increase in MLI. Data presented as mean ± SD. ***p < 0.001. RA, room air; PBS, phosphate-buffered saline.

Representative lung sections stained with hematoxylin and eosin from mice exposed to postnatal RA or hyperoxia and treated with PBS or anti-Tn monoclonal antibody on postnatal day 7 are depicted in **Figure 2A**. The mice reared in hyperoxia and treated with PBS exhibited significantly higher MLI than did those reared in RA and treated with PBS or anti-Tn monoclonal antibody (**Figure 2B**). Treatment with anti-Tn monoclonal antibody significantly decreased the hyperoxia-induced increase in MLI.

### Anti-Tn Monoclonal Antibody Improved Angiogenesis in Hyperoxia-Injured Neonatal Mice

**Figure 3:**
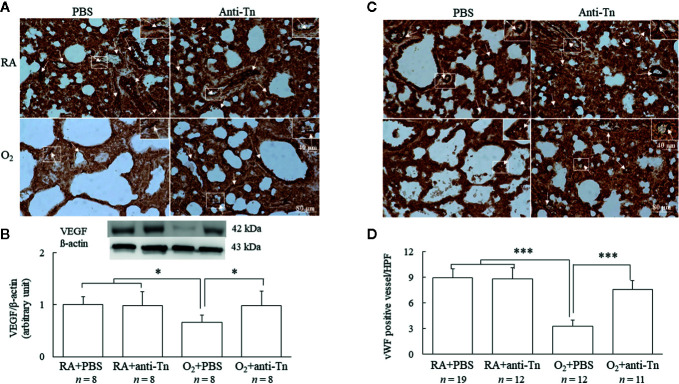
Anti-Tn monoclonal antibody improved angiogenesis in hyperoxia-injured neonatal mice. **(A)** Representative photomicrographs of VEGF staining, **(B)** Western blot and scanning densitometry analyses of VEGF, **(C)** immunohistochemistry of vWF, and **(D)** semiquantitative analysis for vascular density in the lung tissue of 7-day-old mice. Mice reared in hyperoxia and treated with PBS exhibited a significantly decreased VEGF immunoreactivity and vascular density (white arrow), and treatment with anti-Tn monoclonal antibody significantly increased VEGF immunoreactivity and vascular density in these mice compared with PBS-treated mice. The insert shows the positively stained cells and nuclei in more detail. Data presented as mean ± SD. *p < 0.05, ***p < 0.001. RA, room air; PBS, phosphate-buffered saline.

Representative immunohistochemistry of VEGF and vWF are presented in **Figures 3A, C**. The mice reared in hyperoxia and treated with PBS exhibited significantly decreased VEGF immunoreactivity, and treatment with anti-Tn monoclonal antibody significantly increased VEGF immunoreactivity in these mice compared with the PBS-treated mice (**Figure 3A**). Western blot analysis revealed that the mice reared in hyperoxia and treated with PBS exhibited significantly decreased VEGF protein expression, and anti-Tn monoclonal antibody treatment significantly increased VEGF protein expression in these mice compared with the PBS-treated mice (**Figure 3B**). The mice reared in hyperoxia and treated with PBS exhibited significantly lower vascular density than those reared in RA and treated with PBS or anti-Tn monoclonal antibody (**Figures 3C, D** **)**. By contrast, the administration of anti-Tn monoclonal antibody in hyperoxia-exposed mice improved vascular density to normoxic levels.

### Anti-Tn Monoclonal Antibody Reduced a Hyperoxia-Induced Increase in Oxidative Stress

**Figure 4:**
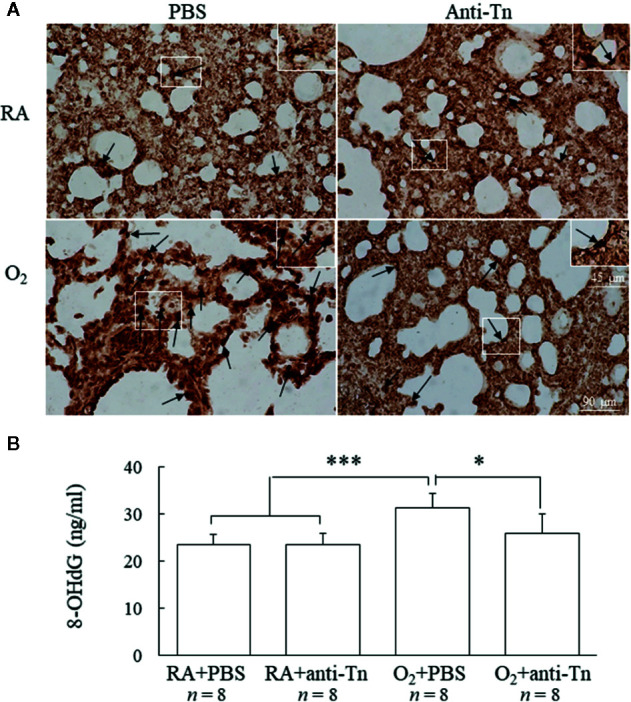
Anti-Tn monoclonal antibody reduced hyperoxia-induced increases in oxidative stress in 7-day-old mice. **(A)** Representative immunohistochemistry of 8-OHdG and **(B)** ELISA of 8-OHdG. Positive staining is indicated in brown (black arrow). Mice reared in hyperoxia and treated with PBS exhibited significantly higher 8-OHdG immunoreactivities and 8-OHdG levels than did those reared in RA and treated with PBS or anti-Tn monoclonal antibody. The insert shows the positively stained cells and nuclei in more detail. Treatment with anti-Tn monoclonal antibody significantly decreased the hyperoxia-induced increase in 8-OHdG immunoreactivities and 8-OHdG levels. Data presented as mean ± SD. *p < 0.05, ***p < 0.001. RA, room air; PBS, phosphate-buffered saline.

Immunohistochemistry was used to detect the oxidative stress marker 8-OHdG, observed as positive staining on nuclei (**Figure 4A**). Mice reared in hyperoxia and treated with PBS exhibited significantly higher 8-OHdG expression than did those reared in RA and treated with PBS or anti-Tn monoclonal antibody (**Figure 4B**). Treatment with anti-Tn monoclonal antibody significantly reduced the hyperoxia-induced increase in 8-OHdG expression.

### Anti-Tn Monoclonal Antibody Reduced Hyperoxia-Induced Increase in NF-*κ*B and Cytokine

**Figure 5:**
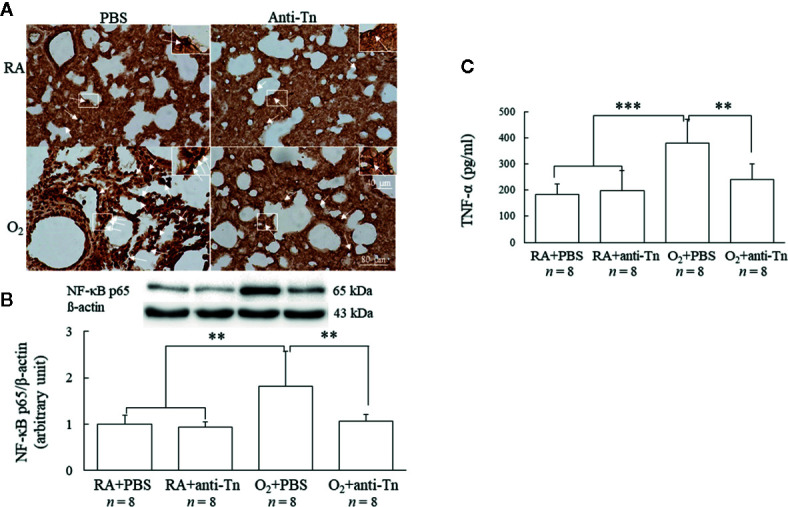
Anti-Tn monoclonal antibody reduced hyperoxia-induced increase in NF-κB and cytokine in 7-day-old mice. **(A)** Representative photomicrographs of NF-*κ*B staining, the insert shows the positively stained cells and nuclei in more detail, **(B)** Western blot and scanning densitometry analyses of NF-*κ*B, and **(C)** TNF-α. The mice reared in hyperoxia and treated with PBS exhibited significantly more intense NF-*κ*B immunohistochemical staining (white arrow), protein expression, and TNF-α expression than did those reared in RA and treated with PBS or anti-Tn monoclonal antibody. Treatment with anti-Tn monoclonal antibody significantly decreased the hyperoxia-induced increase in NF-*κ*B and TNF-α expression. Data are presented as mean ± SD. **p < 0.01, ***p < 0.001. RA, room air; PBS, phosphate-buffered saline.

Representative immunohistochemistry and Western blot and scanning densitometry analyses of NF-*κ*B are presented in **Figures 5A, B**. Immunoreactivity of NF-*κ*B was mostly observed in the cytoplasm and nuclei of alveolar macrophages but was also detected in a few alveolar epithelial cells (**Figure 5A**). The lung sections of the O_2_ + PBS group exhibited more intense NF-*κ*B immunohistochemical staining than those of the RA + PBS and RA + anti-Tn monoclonal antibody groups. Anti-Tn monoclonal antibody treatment significantly reduced the hyperoxia-induced increase in NF-*κ*B immunoreactivity. The O_2_ + PBS group exhibited significantly higher NF-*κ*B protein expression than the RA + PBS and RA + anti-Tn monoclonal antibody groups (**Figure 5B**). Anti-Tn monoclonal antibody treatment significantly reduced the hyperoxia-induced increase in NF-*κ*B protein expression.

The mice reared in hyperoxia and treated with PBS exhibited a significantly higher tumor necrosis factor (TNF)-α level than those reared in RA and treated with PBS or anti-Tn monoclonal antibody (**Figure 5C**). Furthermore, the mice reared in hyperoxia and treated with anti-Tn monoclonal antibody exhibited a significantly lower TNF-α level than those treated with PBS.

## Discussion

Our *in vivo* model demonstrated that hyperoxia exposure during the first 7 days after birth arrested lung and vascular development and induced lung injury, as evidenced by increased MLI and cytokine expression and decreased vascular density. Transforming growth factor-*β*-neutralizing and connective tissue growth factor antibodies have been reported to attenuate hyperoxia-induced lung injury in neonatal rodents (Nakanishi et al., 2007; Alapati et al., 2011; Deng et al., 2019). Anti-Tn monoclonal antibody treatment improved alveolarization and angiogenesis in the lungs of hyperoxia-injured newborn mice, as demonstrated by a reversal of the hyperoxia-induced increase in MLI and decrease in VEGF and vascular density. The improvement of lung development was associated with a reduction in oxidative stress, NF-*κ*B expression, and cytokine expression. Previous studies also revealed that caffeine and iloprost improves hyperoxia-induced lung injury through reduction of oxidative stress and inflammation and attenuation of the endoplasmic reticulum stress and mitochondrial dysfunction (Teng et al., 2017; Olave et al., 2018; Endesfelder et al., 2019). Therefore, we propose that anti-Tn monoclonal antibody improves hyperoxia-induced lung injury in neonatal mice through the suppression of oxidative stress and inflammation, suggesting that anti-Tn antibody may be a promising treatment modality against hyperoxia-induced lung injury. Further studies are needed to elucidate whether the anti-Tn monoclonal antibody effects are mediated by reducing transforming growth factor-β or connective tissue growth factor.

Our study demonstrated that body weights were comparable at birth among the mice before their assignment to the RA or hyperoxia groups. On postnatal day 7, we observed that mice reared in hyperoxia and treated with PBS exhibited significantly lower body weights than did those reared in RA and treated with PBS or anti-Tn monoclonal antibody. These results were different from those of Endesfelder et al. (2019) who found no difference in body weight between postnatal hyperoxia or room air exposure in newborn rats. We speculated that the discrepancy was due to different oxygen exposure time. Anti-Tn monoclonal antibody treatment on postnatal days 2, 4, and 6 preserved the body weight of mice reared in hyperoxia. These results suggest that anti-Tn monoclonal antibody may increase the body weight and improve the health of hyperoxia-exposed newborn mice.

In our previous study, we found that maternal immunization with Tn vaccine increased maternal and neonatal serum anti-Tn antibody titers and attenuated hyperoxia-induced lung injury in newborn rats (Chen et al., 2019). However, immunizing mothers to treat premature birth-related lung diseases is impractical because prematurity is clinically unpredictable and unavoidable. In this study, we evaluated the protective effects of anti-Tn monoclonal antibody on alveolar and vascular development in newborn mice exposed to hyperoxia. We demonstrated that hyperoxia increased oxidative stress and inflammation and led to impaired alveolarization and angiogenesis in neonatal rat lungs (Chen et al., 2019). Consequently, we chose a comparable model and demonstrated hyperoxia affected mice angiogenesis in a similar way. Angiogenesis is a critical element for alveolar development throughout normal lung growth (Jakkula et al., 2000). VEGF is important for angiogenesis during embryonic development because it is a powerful endothelial cell mitogen (McGillick et al., 2016). We demonstrated that hyperoxia exposure during the first 7 days of life reduced vascular density, and anti-Tn monoclonal antibody increased VEGF expression and vascular density in neonatal mice lungs. These results indicate that the administration of anti-Tn monoclonal antibody improved hyperoxia-induced lung injury through increasing VEGF expression.

In neonatal rats, prolonged exposure to hyperoxia increases lung cytokine expression and induces inflammation (Chen et al., 2019). The rise in pro-inflammatory cytokines upregulates cell adhesion molecules, increases chemotactic proteins that attract the inflammatory cells into the lung, and leads to the increase of NF-*κ*B (Shahzad et al., 2016). NF-*κ*B displays both pro-inflammatory and anti-inflammatory effects in lung development (Lawrence et al., 2001). The pro-inflammatory or anti-inflammatory effect of NF-*κ*B plays is dependent on the timing and degree of inhibition (Lawrence et al., 2001; Alvira, 2014). In this study, hyperoxia-reared mice had a significant increase in NF-*κ*B expression, and this was decreased with the administration of anti-Tn monoclonal antibody. Reduced lung cytokine is accompanied the anti-Tn antibody induced reduction in NF-*κ*B activity.

In this study, we examined NF-*κ*B and TNF-α because hyperoxia increases the expression of NF-*κ*B and TNF-α in the lungs of newborn mice and TNF-α activates the NF-*κ*B pathway (Poyet et al., 2000; Chen et al., 2020). 8-OHdG is a DNA base-modified product generated by reactive oxygen species as a marker of oxidative DNA damage and its levels are correlated with other oxidative stress markers (Pérez et al., 2002). The limitation of this study is that we did not measure other oxidative stress markers, such as 3-nitrotyrosine and 8-isoprostane.

In conclusion, this study demonstrated that anti-Tn monoclonal antibody improved alveolar and vascular development induced by hyperoxia in neonatal mice, as indicated by a decrease in MLI and increase in vascular density. The study also revealed that anti-Tn monoclonal antibody does not exert adverse effects on normal neonatal body growth or lung development. The beneficial effects of anti-Tn monoclonal antibody on hyperoxia-induced lung injury are mediated by a decrease in oxidative stress, cytokine expression, and NF-κB activity as well as an increase in VEGF expression. Currently, no effective therapy is available for preventing hyperoxia-induced lung injury. These findings suggest the therapeutic potential of anti-Tn monoclonal antibody for treating hyperoxia-induced lung injury.

## Data Availability Statement

The raw data supporting the conclusions of this article will be made available by the authors, without undue reservation.

## Ethics Statement

The animal study was reviewed and approved by Animal care and experimental procedures were performed in accordance with the guidelines of the Laboratory Animal Care Committee of Taipei Medical University (LAC-2019-0149).

## Author Contributions

Designed and performed the experiments: C-MC and JH. Analysis and interpretation of data: C-MC, JH, H-CC, and CC. Drafted and approved the manuscript: C-MC, JH, H-CC, and CC.

## Funding

This study received grants from the Ministry of Science and Technology in Taiwan (107-2314-B-038-060-MY2).

## Conflict of Interest

Author CC was employed by company Taivital Biopharmaceutical Co. LTD.

The remaining authors declare that the research was conducted in the absence of any commercial or financial relationships that could be construed as a potential conflict of interest..
